# Crosslinking assay to study a specific cargo-coat interaction through a transmembrane receptor in the secretory pathway

**DOI:** 10.1371/journal.pone.0263617

**Published:** 2022-02-10

**Authors:** Javier Manzano-Lopez, Sofia Rodriguez-Gallardo, Susana Sabido-Bozo, Alejandro Cortes-Gomez, Ana Maria Perez-Linero, Rafael Lucena, Antonio Cordones-Romero, Sergio Lopez, Auxiliadora Aguilera-Romero, Manuel Muñiz

**Affiliations:** Department of Cell Biology, University of Seville and Instituto de Biomedicina de Sevilla (IBiS), Hospital Universitario Virgen del Rocío/CSIC/Universidad de Sevilla, Seville, Spain; University of Toronto, CANADA

## Abstract

Intracellular trafficking through the secretory organelles depends on transient interactions between cargo proteins and transport machinery. Cytosolic coat protein complexes capture specific luminal cargo proteins for incorporation into transport vesicles by interacting with them indirectly through a transmembrane adaptor or cargo receptor. Due to their transient nature, it is difficult to study these specific ternary protein interactions just using conventional native co-immunoprecipitation. To overcome this technical challenge, we have applied a crosslinking assay to stabilize the transient and/or weak protein interactions. Here, we describe a protocol of protein crosslinking and co-immunoprecipitation, which was employed to prove the indirect interaction in the endoplasmic reticulum of a luminal secretory protein with a selective subunit of the cytosolic COPII coat through a specific transmembrane cargo receptor. This method can be extended to address other transient ternary interactions between cytosolic proteins and luminal or extracellular proteins through a transmembrane receptor within the endomembrane system.

## Introduction

In eukaryotic cells, one-third of the proteome is synthesized in the endoplasmic reticulum (ER) and subsequently sorted during transport through the secretory pathway for delivery to their proper cellular destinations including the extracellular space, the plasma membrane, the endo-lysosomal system, or the secretory granules in specialized cells. To initiate the secretory pathway, correctly folded and assembled newly synthesized secretory proteins are segregated in the ER from immature and resident proteins, and selectively incorporated into protein-coated membrane vesicles that transport them from the ER to the Golgi [1]. These vesicles are made at specific regions of the ER membrane called ER exit sites (ERES) by the sequential assembly of the cytosolic COPII coat proteins. For efficient uptake into nascent COPII vesicles at ERES, a variety of luminal cargo requires transmembrane adaptors or receptors that physically link each specific cargo in the lumen with the COPII coat in the cytosol [2–4]. These cargo proteins are escorted by their receptors into COPII vesicles to the Golgi, where they are released to continue their transport through the secretory pathway to their destination. The empty receptors then interact with the cytosolic COPI coat to be transported back from the Golgi to the ER in COPI-coated vesicles and thus initiate a new round of anterograde transport. Therefore, a better understanding of protein sorting in the early secretory pathway requires the detection and analysis of specific interactions of transmembrane receptors with cargo proteins and different vesicle coats. The fact that many of these interactions are transient and unstable implies that they may not be detected just with conventional methods such as the native co-immunoprecipitation. To solve this methodological problem, transient and/or weak protein interactions can be stabilized by chemical crosslinking before co-immunoprecipitation [5].

**Fig 1:**
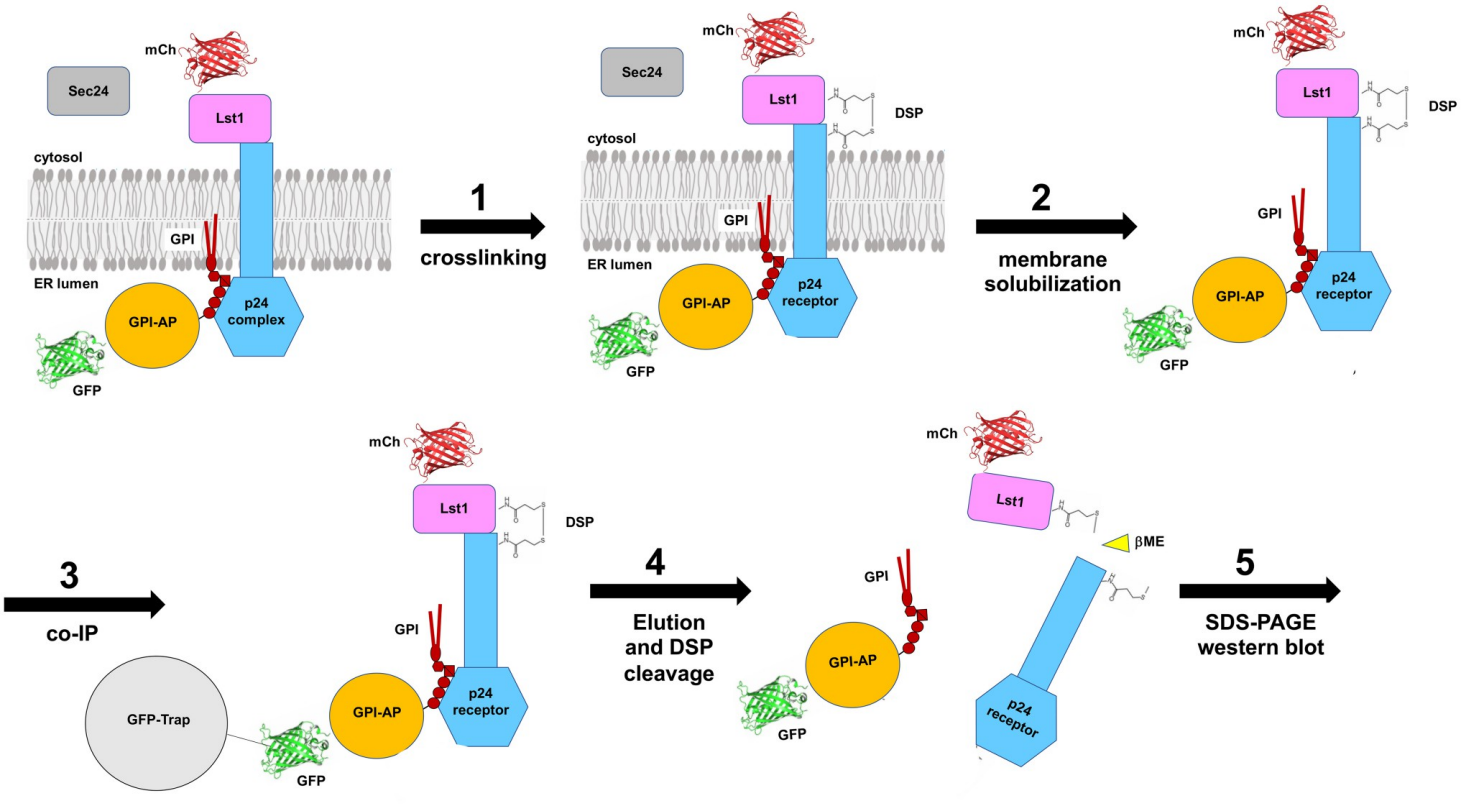
Experimental workflow for the crosslinking assay. Extracts of cells expressing the specialized COPII cargo-binding isoform Lst1 tagged with mCherry (mCh) and a GPI-AP tagged with GFP are treated with the crosslinker DSP to capture transient protein-protein interactions (1). After quenching the crosslinking reaction, the membrane fraction is solubilized with the mild non-ionic detergent digitonin to preserve the direct interaction between the GPI-AP and the transmembrane p24 receptor (2). The GPI-AP-GFP is then immunopurified by the GFP-trap system (3). After breaking the disulfide bond in the spacer of DSP under reducing conditions (4), co-immunopurified crosslinked proteins are separated by SDS-polyacrylamide gel electrophoresis (SDS-PAGE) (5) and analyzed by Western blotting (6).

We have used a specialized protocol of protein crosslinking and co-immunoprecipitation to address the cargo receptor-dependent ER export of glycosylphosphatidylinositol (GPI)-anchored proteins (GPI-APs), which are secretory proteins attached by the glycolipid GPI anchor to the luminal leaflet of the ER membrane [6]. Due to this luminal topology, newly synthesized GPI-APs require a transmembrane cargo receptor that connects them with the cytosolic COPII coat for ER export. Previous studies from our laboratory and others suggested that the p24 protein complex, composed of type I transmembrane p24 proteins, can act as a specific cargo receptor for GPI-APs [7–9]. It was hypothesized that the p24 receptor would link GPI-APs to Lst1, a specialized isoform of the major COPII coat cargo binding subunit Sec24, to be actively packaged into nascent COPII vesicles [5,7,10]. However, testing this hypothesis was technically challenging since it involved the detection of a ternary protein interaction. Indeed, by using native co-immunoprecipitation with a mild detergent like digitonin we could preserve the direct interaction of the p24 receptor with GPI-APs but not with the COPII coat [5]. To capture this ternary protein interaction, we applied a crosslinking assay before native co-immunoprecipitation to an extract from yeast cells expressing a GPI-AP tagged with GFP (GPI-AP-GFP) and the COPII subunit Lst1 tagged with mCherry (Lst1-mCherry) [5]. The GPI-AP-GFP was expressed in a *CEN* plasmid, whereas the Lst1-mCherry fusion gene was integrated and expressed in the genome using an integrative plasmid to achieve a uniform co-expression across the population of yeast cells. Yeast cells endogenously expressing the GPI-AP-GFP and Lst1-mCherry were broken by glass beads and the lysate was incubated with the membrane-permeable crosslinker dithiobis(succinimidylpropionate) (DSP). DSP contains an amine-reactive *N*-hydroxysuccinimide (NHS) ester at each end of a spacer arm with a cleavable disulfide bond [11]. NHS esters can react with primary amines in the side chain of lysine residues and the N-termini of proteins, thereby forming stable amide bonds that crosslink interacting proteins [11]. The crosslinking reaction was finally quenched by the addition of glycine and differentially centrifuged to generate an enriched ER membrane fraction that increases the specific recovery of proteins present in the ER. The ER membrane fraction was solubilized with the mild non-ionic detergent digitonin to preserve the direct interaction between the GPI-AP and the transmembrane p24 receptor. The GPI-AP-GFP was then immunopurified by the GFP-trap system. After breaking the disulfide bond in the spacer of DSP under reducing conditions, co-immunopurified crosslinked proteins were separated by SDS-polyacrylamide gel electrophoresis (SDS-PAGE) and analyzed by Western blotting (Fig 1). By using this method, we show that the transmembrane p24 protein complex receptor specifically connects GPI-APs in the ER lumen with the specialized COPII subunit Lst1 in the cytosol [12]. Therefore, this method exposes the discriminatory capacity of the luminal cargo to bind specific coats through their interaction with the cargo receptor. Using the crosslinking we could address basic questions of the secretory pathway as the importance of the cargo maturation in the recruitment of the appropriate coat or the influence of membrane lipids in the specific receptor coat recruitment [5,13]. Moreover, this crosslinking assay can be potentially extended to study any kind of ternary interaction between luminal or extracellular proteins and cytosolic proteins through a transmembrane receptor or adaptor within the secretory and endocytic pathways.

## Materials and methods

The protocol described in this peer-reviewed article is published on protocols.io dx.doi.org/10.17504/protocols.io.b3d9qi96 and is included for printing as S1 Protocols with this article.

### Expected results

**Fig 2:**
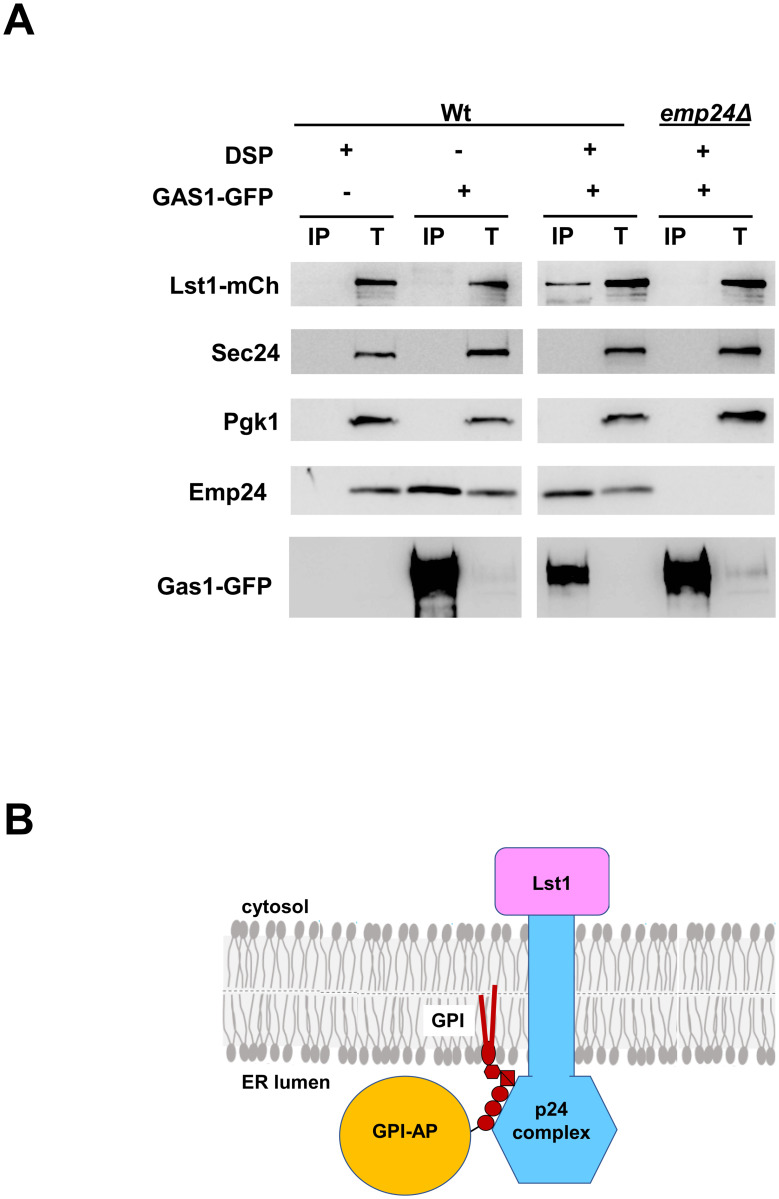
Crosslinking assay between the GPI-AP Gas1-GFP and the specialized COPII cargo-binding subunit Lst1-mCh. (A) Extracts of wild-type and *emp24*Δ deletion strains expressing Gas1-GFP, and Lst1-mCh were incubated with (+) and without (-) DSP, solubilized, and immunoprecipitated with the GFP-Trap system, followed by immunoblotting with anti-mCherry, anti-Sec24, anti-Pgk1, anti-Emp24, or anti-GFP antibodies. T represents 1% of the solubilized input material. The experiment was repeated three times. (B) Graphical representation of the model based on the crosslinking assay presented here. The p24 complex connects GPI-Aps preferentially with the specialized COPII cargo-binding subunit Lst1, but not with the major COPII cargo-binding subunit Sec24.

GPI-APs are lipid-linked secretory proteins. Once attached to the GPI anchor in the ER lumen, newly synthesized GPI-APs are exported from the ER for delivery to the plasma membrane along the secretory pathway [6,14]. Efficient ER export of GPI-APs requires both the p24 transmembrane protein complex and the specialized COPII subunit Lst1 [7]. The p24 proteins are conserved and abundant type I transmembrane proteins with a luminal domain and a short cytoplasmic tail that harbors COPII and COPI coat binding signals [15]. The p24 proteins are assembled in a heteromeric complex that cycles between the ER and Golgi compartments. Lst1 is an isoform of the COPII cargo-binding subunit Sec24 required to export from the ER a specific subset of secretory cargo proteins including GPI-APs [7,10,16]. Because the cytosolic tail of the p24 proteins can bind to Lst1 in addition to Sec24, it was proposed that the p24 complex might specifically connect GPI-APs to Lst1 [10]. Using the method described here, we addressed this hypothesis and we could show that the mature GPI-AP specifically recruits the cytosolic Lst1 coat through its interaction with the p24 complex [12]. As a proof of concept and to analyze the extent of the association between GPI-APs and Lst1, we studied the specific recruitment of Lst1 by another GPI-AP, Gas1. For this purpose, an extract of wild-type strain expressing the GPI-AP Gas1 tagged with GFP and Lst1-mCherry was incubated with and without the membrane-permeable crosslinker DSP, solubilized, and immunoprecipitated by the GFP-Trap system, followed by immunoblotting with different antibodies, including anti-mCherry, anti-p24, or anti-GFP antibodies. As seen in Fig 2A, cytosolic Lst1-mCherry was co-immunoprecipitated by luminal Gas1-GFP in wild-type cells only in the presence but not in the absence of the DSP crosslinker. In addition to Lst1-mCherry, the p24 complex component Emp24 was also co-immunoprecipitated by Gas1-GFP but, in this case, independently of the presence of the crosslinker because, as previously observed, the use of the mild detergent digitonin preserves the more stable interaction between the p24 complex and GPI-APs [5,7]. As a negative control for the co-immunoprecipitation assay, we applied the same crosslinking protocol to an extract of wild-type strain expressing no Gas1-GFP. As expected, neither Lst1-mCherry nor Emp24 was immunoprecipitated by the GFP-Trap beads. We also examined whether the p24 transmembrane protein complex mediates the observed interaction between Lst1-mCherry in the cytosol and Gas1-GFP in the ER lumen. We then used the *EMP24* deletion to destabilize the other p24 proteins of the complex, leading to a complete loss of the p24 complex function [17]. As shown in Fig 2A, Lst1-mCherry was not crosslinked to Gas1-GFP in the *emp24*Δ deletion strain. This result indicates that the p24 transmembrane complex connects GPI-APs in the ER lumen to Lst1 in the cytosol. To address the specificity or selectivity of the binding between Gas1-GFP and Lst1-mCherry mediated by the p24 complex, two internal negative binding controls were carried out. First, we showed that the unrelated cytosolic protein Pgk1 was not co-immunoprecipitated by Gas1-GFP after crosslinking. We also provided additional internal negative control for the binding specificity of Lst1-mCherry by probing against Sec24, the major COPII cargo binding isoform. Interestingly, the cytosolic tails of the p24 proteins can directly recognize a specific binding site present in both COPII isoforms, Sec24 and Lst1 [10]. In agreement, we have previously shown that Sec24 and Lst1 can be crosslinked to Emp24-GFP [5,13]. However, Lst1 is specifically required for GPI-AP ER export of [7], suggesting that p24 could selectively connect Lst1 but not Sec24 to GPI-APs despite that p24 can interact with both COPII subunits. This possibility was fully supported by the presented crosslinking experiment (Fig 2A), since only Lst1-mCherry but not Sec24 was co-immunoprecipitated by Gas1-GFP in a p24 dependent manner. Therefore, Sec24 represents, in addition to Pgk1, a good internal negative binding control that shows that the presented assay can discriminate the specificity of p24 for Lst1 upon binding of GPI-APs.

Furthermore, from the presented crosslinking experiment, we cannot exclude that GPI-AP cargo, p24, and Lst1 may interact indirectly through other bridging proteins. However, it is highly likely that they interact directly to form a ternary complex because our and others previous studies have shown direct interaction of p24 with the GPI-AP Gas1 and the presence of a specific site in Lst1 for direct binding of p24 cytosolic tails [8,10]. Taken together these results provide evidence that the p24 complex functions as a selective transmembrane adaptor or cargo receptor by connecting GPI-APs in the ER lumen with the specific COPII protein Lst1 instead of with the major COPII cargo binding subunit Sec24, for ER export in specialized COPII vesicles (Fig 2B).

## Supporting information

S1 Fig (TIF)

S1 Protocols (PDF)
