# Supplementary figures and images for: Crosslinking assay to study a specific cargo-coat interaction through a transmembrane receptor in the secretory pathway
Source: PLoS One. 2022 Feb 10;17(2):e0263617. doi: 10.1371/journal.pone.0263617 (PMC8830656; doi:10.1371/journal.pone.0263617)

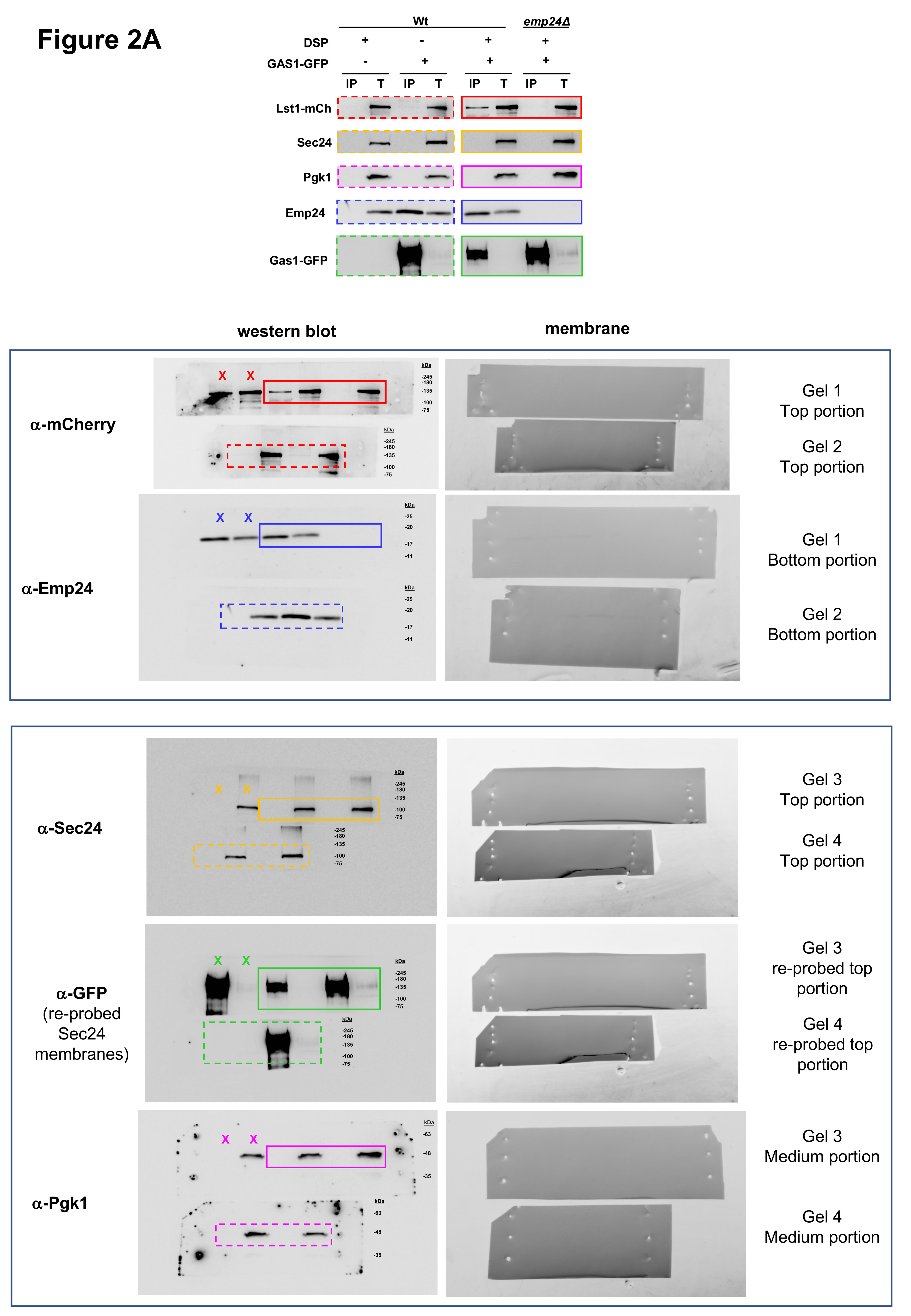

Supplement: S1 Fig — (TIF) [file pone.0263617.s001.tif]
