# Supplementary material for: Crosslinking assay to study a specific cargo-coat interaction through a transmembrane receptor in the secretory pathway
Source: PLoS One. 2022 Feb 10;17(2):e0263617. doi: 10.1371/journal.pone.0263617 (PMC8830656; doi:10.1371/journal.pone.0263617)
Supplement: S1 Protocols — (PDF) [file pone.0263617.s002.pdf]

Crosslinking assay to study a specific cargo-coat interaction through a transmembrane receptor in the secretory pathway

Javier Manzano-Lopez†*[1], Sofia Rodriguez-Gallardo†[1], Susana Sabido-Bozo†[1], Alejandro Cortes-Gomez[1], Ana Maria Perez-Linero[1], Rafael Lucena[1], Antonio Cordones-Romero[1], Sergio Lopez[1], Auxiliadora Aguilera-Romero*[1], Manuel Muñiz*[1]

[1]Department of Cell Biology, University of Seville and Instituto de Biomedicina de Sevilla (IBiS), Hospital Universitario Virgen del Rocío/CSIC/Universidad de Sevilla, 41012, Seville, Spain.

1

Manuel Muñiz

Intracellular trafficking through the secretory organelles depends on transient interactions between cargo proteins and transport machinery. Cytosolic coat protein complexes capture specific luminal cargo proteins for incorporation into transport vesicles by interacting with them indirectly through a transmembrane adaptor or cargo receptor. Due to their transient nature, it is difficult to study these specific ternary protein interactions just using conventional native co-immunoprecipitation. To overcome this technical challenge, we have applied a crosslinking assay to stabilize the transient and/or weak protein interactions. Here, we describe a protocol of protein cross-linking and co-immunoprecipitation, which was employed to prove the indirect interaction in the endoplasmic reticulum of a luminal secretory protein with a selective subunit of the cytosolic COPII coat through a specific transmembrane cargo receptor. This method can be extended to address other transient ternary interactions between cytosolic proteins and luminal or extracellular proteins through a transmembrane receptor within the endomembrane system.

Javier Manzano-Lopez†*, Sofia Rodriguez-Gallardo†, Susana Sabido-Bozo†, Alejandro Cortes-Gomez, Ana Maria Perez-Linero, Rafael Lucena, Antonio Cordones-Romero, Sergio Lopez, Auxiliadora Aguilera-Romero*, Manuel Muñiz* . Crosslinking assay to study a specific cargo-coat interaction through a transmembrane receptor in the secretory pathway. **protocols.io** https://protocols.io/view/crosslinking-assay-to-study-a-specific-cargo-coat-b3d9qi96

Cross-linking, DSP, COPII, receptor, p24 complex, GPI-APs

Jan 01, 2022

Jan 03, 2022

56481

Recipes

Growth media

1. SD agar plates: synthetical minimal medium containing 2% glucose, 0.67% yeast nitrogen base, 0.5% ammonium sulfate, and 2% bacteriological agar, supplemented with the appropriate nutritional requirements (0.012% except for adenine 0,03%) to complement auxotrophies.
2. SD medium: synthetical minimal medium containing 2% glucose, 0.67% yeast nitrogen base and 0.5% ammonium sulphate, supplemented with the appropriate nutritional requirements (0.012% except for adenine 0,03%) to complement auxotrophies.
3. YPD medium: rich medium containing 2% glucose, 1% yeast extract, and 2% peptone, supplemented with 0.2% adenine and uracil.

Buffers

1 One-step buffer: 0.2 N lithium acetate, 40% PEG 3350, 100 mM DTT pH 5.0
2. 1000x PI (Protease inhibitor cocktail): antipain 0.1%, leupeptin 0.1% and pepstatin 0.1% in DMSO
3. 100x PMSF: 100mM phenylmethylsulphonyl fluoride in isopropanol
4. B88: 20 mM HEPES, pH 6.8, 150 mM KOAc, 250 mM sorbitol, 5 mM $Mg(OAc)_2$, 1 mM PMSF, 1x PI
5. PBS (Phospate Buffered Saline): 137 mM NaCl, 2.7 mM KCl, 8 mM Na_2HPO_4, and 2 mM KH_2PO_4 pH 7.4, 1 mM PMSF, 1x PI
6. PBS-D 0,2%: PBS containing 0,2% (w/v) digitonin
7. PBS-D 1%: PBS containing 1% (w/v) digitonin
8. PBS-D 5%: PBS containing 5% (w/v) digitonin
9. 10x DSP fresh stock solution (10mM DSP in DMSO anhydrous). Dissolve DSP in DMSO anhydrous immediately before use.
10. 10x glycine stock solution: 250 mM glycine pH 7.5
11. SB 2x (Sample buffer): 4% SDS, 20% glycerol, 10% 2-mercaptoethanol, 0.004% bromophenol blue and 0.125 M Tris HCl, pH 6.8. Add 2-mercaptoethanol immediately before use.
12. TBS (Tris-Buffered Saline): 20 mM Tris-HCl pH8, 150 mM NaCl
13. TBS-T; TBS containing 1% Tween-20
14. TBS-T+ 5% milk: TBS-T containing 5% skimmed milk powder
15. TBS-T+ 5% milk + 0.01% sodium azide: TBS-T containing 5% skimmed milk powder and 0.01% (v/v) sodium azide
16. TBS-T + 100 mM 2-mercaptoethanol + 2% SDS
17. Stripping buffer: 25 mM Glycine-HCl pH 2, 1% SDS

Equipment

1. Incubator shaker (Eppendorf, model M1299-0092)
2. Micropipettes (Gilson, Pipetman, models: P20, P200, P1000)
3. Spectrophotometer or microplate reader (any brand with 600 nm wavelength) or equipment to count cell density
4. Bead beater (Fastprep, MP Biomedicals, model: Fastprep-24).
5. Centrifuge (Thermo scientific, model: Heraeus Fresco 17; Eppendorf, model: centrifuge 5818R)
6. Orbital wheel (any brand)
7. Thermoshaker (Thermo Fisher, model 13687711)
8. Electrophoresis system (Mini PROTEAN, Bio-Rad catalog number: 1658005)
9. Power supply (Bio-Rad, catalog number: 1645052)
10. Western Blot Transfer system (Mini Trans-Blot Electrophoretic Transfer Cell, Bio-Rad catalog

number: 1703930)
11. Orbital shaker (any brand)
12. Chemiluminescence Imaging System (ChemiDoc MP Imaging System, Bio-Rad catalog number: 17001402)

Materials

1. Budding yeast cells (W303) genomically expressing Lst1-mCherry. This yeast strain can be obtained upon request from our laboratory.
2. Centromeric plasmid expressing GFP-tagged Gas1 under control of its own promoter (pRS416-GAS1-GFP) was a generous gift from Laura Popolo (University of Milan) (E. Rolli et al. 2009, *Mol. Biol. Cell* 20, 4856-4870).
3. Toothpicks sterilized before use (any brand)
4. Yeast extract (Pronadisa catalog number: 1702.00)
5. Peptone (Panreac catalog number: A2210,0500)
6. Glucose (Panreac catalog number: 131341.0914)
7. Yeast nitrogen base without amino acids (Difco Laboratories, catalog number: 291940)
8. Nutritional supplements:
    a. Uracil SIGMA catalog number: U0750
    b. L-Leucine SIGMA catalog number: L8000
    c. L-Tryptophan SIGMA catalog number: T0254
    d. Adenine SIGMA catalog number: A8626
    e. L-Histidine SIGMA catalog number: H8000
    f. L(+)-Lysine SIGMA catalog number: L5501
    g. L-Methionine SIGMA catalog number: M9625)
9. Agar (Oxoid catalog number: LP0011)
10. Lithium acetate dihydrate (Sigma catalog number: L6883)
11. Polyethylene glycol (PEG) 3350 (USP catalog number:1546547)
5. DL-Dithiothreitol (DTT) (Sigma catalog number: D0632)
12. Deoxyribonucleic acid, single-stranded from salmon testes (Salmon Sperm DNA) (Sigma catalog number: D9156)
13. 15 ml conical tubes (any brand)
14. 12 ml tube (any brand)
15. 1.5 ml microcentrifuge tubes (any brand)
16. 50 ml conical tubes (any brand)
17. 1.5 ml screw-cap microcentrifuge tubes (any brand)
18. Glass beads (SIGMA catalog number: G9268-500G)
19. 1.5 ml UltraClear microcentrifuge tubes (Axygen catalog number: MCT-175-L-C)
20. DSP (dithiobis[succinimidylpropionate]) Lomant's Reagent (Thermo Fisher Scientific catalog number: 22585)
21. DMSO (Dimethyl sulfoxide) anhydrous (Sigma catalog number: 276855-100ML)
22. Digitonin (Panreac AppliChem catalog number: A1905,0005)
23. Naked Bab Agarose beads (ChromoTek, catalog number: bab-20)
24. GFP-Trap Agarose beads (ChromoTek, catalog number: gta-20)
25. Protease Inhibitors (PI):
    a. Antipain (SIGMA catalog number: A6191)
    b. Leupeptin (SIGMA catalog number: L2023)
    c. Pepstatin A (SIGMA catalog number: P4265)
26. PMSF (Amresco catalog number: 0754-5G)
27. Glycine (Amresco number catalog: 0167-1KG)
28. PBS (Phosphate Buffered Saline) (Amresco catalog number: 0780-50L)
29. Tris (Amresco catalog number: 0497-1KG)
30. NaCl (Panreac catalog number: 131659.1211)
31. EDTA (Amresco catalog number: 0322-500G)

32. HCl 5mol/l (Panreac catalog number: 182109.1211)
33. Acetic Acid Glacial (Panreac catalog number: 141008.1211)
34. 10% Mini-PROTEAN® TGX™ Precast Protein Gels, 10-well, 30 µl (Bio-Rad catalog number: 4561023)
35. 2-Mercaptoethanol (Merck catalog number: M3148-25ML)
36. Precision Plus Protein Dual Color Standards (Bio-Rad catalog number 1610374)
37. Nitrocellulose membrane (Amersham™ Protran® Western blotting membranes, nitrocellulose, Merck catalog number: GE10600002)
38. Ponceau staining solution (Ponceau S solution, Sigma-Aldrich catalog number P7170)
39. Tween-20 (polyoxyethylene sorbitan) (Amresco catalog number: 0777-1L)
40. Skimmed milk powder (any brand)
41. Sodium azide (Sigma Aldrich number catalog: S2002)
42. RFP antibody [6G6] (recognizes mCherry tag) (ChromoTek, catalog number: AB_2631395)
43. Non-commercial antibodies to Sec24, Emp24, Pgk1, and GFP were generous gifts from the Riezman lab (University of Geneve, Switzerland).
44. HRP-conjugated secondary antibodies (Goat anti-Rabbit IgG (H+L) Secondary Antibody, HRP, Invitrogen number catalog: 31466)
45. ECL western blot substrates (SuperSignal™ West Pico Plus, Thermo Scientific catalog number: 34580, and SuperSignal™ West Atto, Thermo Scientific catalog number: A38554)

Yeast transformation and culture

1 Transform the yeast strain genomically expressing Lst1-mCherry with a centromeric plasmid expressing GFP-tagged Gas1 under control of its own promoter (pRS416-GAS1-GFP).

1.1 Grow a 5 ml overnight culture of the yeast strain to be transformed at 24ºC with shaking at 250 rpm.

1.2 The next day, collect 5 x10^7 cells into a 15 ml tube and centrifuge at 3,000 x g for 5 min at room temperature.

1.3 Discard the supernatant, resuspend the cell pellet in 1 ml of sterile dH_2O, transfer into a 1.5 ml tube, and centrifuge at 3,000 x g for 5 min at room temperature.

1.4 Discard the supernatant and resuspend the cell pellet in 100 µl with “One-step” buffer.

1.5 Add 1 µg of plasmid DNA (pRS416-GAS1-GFP) and 50 µg of boiled salmon sperm DNA. Briefly vortex.

1.6 Incubate 30-40 min at 45°C and plate on appropriate selection media (SC-URA).

1.7 Note: Carry out a negative control sample without DNA plasmid.

2 Pick up a transformed colony and streak it on an SC-URA agar plate and incubate them at 24°C for 2-3 days.

3 Inoculate transformed yeast cells in 3 ml of SC-URA in a 12 ml sterile tube and grow them to the early-to-mid logarithmic phase at 24°C with shaking at 250 rpm.

4 Dilute yeast cells in 200 ml of YPD medium and grow them overnight at 24ºC with shaking at 250 rpm until reaching 1.5 x 10^7 cells/ml. Note: YPD medium is required for correct cell surface expression of Gas1-GFP.

5 Collect 240 x 10^7 cells per sample condition and centrifuge at 3000 x g for 5 min at 4°C.

6 Discard the supernatant and resuspend by vortexing the cell pellet in 1.5 ml of B88 buffer precooled at 4°C. Transfer the cell suspension to a 2 ml screw-cap tube.

7 Centrifuge at 13,000 x g for 1 min at 4°C, discard the supernatant and freeze the pellet at -80ºC.

Cell lysis

8 Quick thaw the cell pellets and immediately place them on ice.

9 Resuspend each cell pellet with 1.5 ml of prechilled B88 buffer.

10 Add 300 µl of glass beads per tube and lyse mechanically the cells using a bead beater system. For Fastprep device: 3 pulses of 20 s at 5 m/s. Rest on ice for 3 min between pulses.

11 Spin down glass beads and cell debris at 1000 x g for 10 min at 4°C.

12 Collect 1 ml of supernatant into a 1.5 ml ultraclear tube.

Cross-linking

13 During the 10 min centrifugation at 4°C at 1000 x g (step 11), prepare a fresh 10x DSP stock solution (10mM DSP in DMSO anhydrous).

14 Add 110 µl of 10x DSP stock solution to the cleared cell extract (step 12) and incubate for 20 min at 20ºC in a thermoblock.

15 Quench the cross-linking reaction by adding 150 µl of 10x glycine stock solution and incubate for 5 min at 20ºC.

16 Spin down cellular membranes at 13,000 x g for 15 min at 4°C to enrich the pellet with ER membranes.

17 Discard the supernatant and resuspend each membrane pellet in 1 ml of PBS at 4ºC.

co-immunoprecipitation of cross-linked proteins

18 Centrifuge the cell suspension at 17,000 x g for 20 min at 4°C to remove insoluble membranes.

19 Transfer the supernatant to a 1.5 ml ultraclear tube and add 100 µl of a 15% solution of naked agarose beads (Bab beads) and rotate for 1 h at 4°C to remove unspecific protein binding.

20 Centrifuge at 5,000 x g for 30 s and transfer the supernatant to a 1.5 ml ultraclear tube.

21 Save a 20 µl aliquot of each supernatant for the analysis of protein expression. These will be the total lysate input samples.

22 Add 100µl of GFP-Trap agarose beads (30% slurry) to the remainder of the supernatant (step 21) and rotate overnight at 4°C.

23 The next day centrifuge at 5,000 x g for 30 s and remove supernatant.

24 Resuspend the beads pellet with 1 ml of PBS-D 1% and transfer to a new 1.5 ultraclear tube.

25 Centrifuge at 5,000 x g for 30 s and remove the supernatant.

26 Add 1 ml PBS-D 0.2%. Repeat steps 25 and 26 twice more.

27 Centrifuge at 5,000 x g for 30 s and remove the supernatant. Centrifuge again to remove the remaining liquid and dry the beads using a white gel-loading micropipette tip.

28 Elute proteins from the beads and dissociate the crosslinked protein complexes by adding 40µl of 1x SDS-PAGE sample buffer containing fresh 5% 2-mercaptoethanol at 95°C for 10 min.

29 Vortex and spin down the beads to the maximum speed at room temperature for 1 min and collect the supernatant (IP sample).

30 Add 20 µl of 2x SDS-PAGE sample buffer containing fresh 5% 2-mercaptoethanol to the tubes containing 20 µl of the total lysate input (T sample) from step 21, vortex and incubate at 95°C for 10 min.

Western blotting

31 Load IP samples (20 µl) with their respective T samples (10 µl) onto a 10% acrylamide gel. Separate the proteins by SDS-PAGE gel electrophoresis and transfer them to a nitrocellulose membrane.

32 To check protein transfer, stain the nitrocellulose membrane in Ponceau Staining Solution for 3 min and wash with distilled water.

33 Block the membranes in TBS-T + 5% milk for 1 h at room temperature with shaking.

34 Blot the crosslinked proteins with anti-RFP (1:500), anti-Sec24 (1:1000), anti-Emp24 (1:1000), anti-GFP (1:500), and anti-Pgk1 (1:5000) antibodies sequentially with membrane stripping between blots to detect Lst1-mCherry (~132 kD), Sec24 (~104 kD), Pgk1 (~45 kD), and Gas1-GFP (~135 kD) respectively. Alternatively, since all these antibodies are highly specific, the total number of strippings can be reduced by cutting the membrane horizontally after protein transfer and probing each separate membrane portion with the different antibodies. In this particular case, cut the membrane horizontally at 75 kD and 25 kD prestained protein markers in three portions. Probe sequentially the top portion for Lst1-mCherry, Sec24, and Gas1-GFP with membrane stripping between the blots. Probe medium and bottom portions for Pgk1 and Emp24 respectively. In addition to reducing the total number of strippings, this also saves limited primary antibodies.

35 Incubate the membranes in primary antibody diluted in TBS-T + 5% milk 1 h at room temperature or overnight at 4°C, with shaking.

36 Wash the membranes in TBS-T + 5% milk, 5x 5 min.

37 Incubate the membranes in HRP-conjugated secondary antibody diluted in TBS-T + 5% milk 1 h at room temperature with shaking.

38 Wash the membranes in TBS-T 5x 5 min.

39 Visualize crosslinked proteins by using enhanced chemiluminescence (ECL) detection through the Bio-Rad ChemiDoc instrument.

Membrane stripping for antibody reprobing

40 Wash the membranes 3x 3 min in TBS-T.

41 Incubate the membranes for 15 min at room temperature in TBS-T + 100 mM 2-mercaptoethanol + 2% SDS.

42 Wash the membranes 3x 5 min in TBS-T.

43 Incubate the membranes for 30 min at room temperature in stripping buffer.

44 Wash the membranes 6x 5 min in TBS-T. The stripped membranes are now ready for reprobing with another antibody (step 35). The stripped membrane can be also kept overnight in TBS-T + 5% milk + 0.01% sodium azide at 4ºC. In this case, wash the membrane 3x 3 min in TBS-T before reproving with the antibody.

45 Block the membranes in TBS-T + 5% milk for 1 h at room temperature with shaking.

46 Reprobe the stripped membranes with another antibody (step 36).

Controls for binding specificity

47 Carry out appropriate binding specificity controls:
a) Wild-type yeast cells expressing no Gas1-GFP.
b) Without crosslinker.
c) Detection of a non-specific protein such as the cytosolic protein Pgk1.
